# Distribution and Host Selection of Tropical Rat Mite, *Ornithonyssus bacoti*, in Yunnan Province of Southwest China

**DOI:** 10.3390/ani11010110

**Published:** 2021-01-07

**Authors:** Peng-Wu Yin, Xian-Guo Guo, Dao-Chao Jin, Rong Fan, Cheng-Fu Zhao, Zhi-Wei Zhang, Xiao-Bin Huang, Ke-Yu Mao

**Affiliations:** 1 Institute of Entomology, Guizhou University, and the Provincial Key Laboratory for Agricultural Pest Management in Mountainous Region, Guiyang 550025, China; pengwuyin@vip.163.com (P.-W.Y.); jindaochao@163.com (D.-C.J.); 2 Vector Laboratory, Institute of Pathogens and Vectors, Yunnan Provincial Key Laboratory for Zoonosis Control and Prevention, Dali University, Dali 671000, China; fanfanlook1@outlook.com (R.F.); zchengf2020@163.com (C.-F.Z.); dalizzw@aliyun.com (Z.-W.Z.); huangxb633@nenu.edu.cn (X.-B.H.); 15887382535@163.com (K.-Y.M.)

**Keywords:** Acari, gamasid mite, *Ornithonyssus bacoti*, distribution, host selection, Yunnan, China

## Abstract

**Simple Summary:**

The tropical rat mite (*Ornithonyssus bacoti*) is a transmission vector of rickettsia pox and a potential vector of hemorrhagic fever with renal syndrome (HFRS). This article reports the distribution and host selection of *O. bacoti* in Yunnan Province of Southwest China. The original data came from the investigations in 39 counties of Yunnan. The prevalence (*P_M_*), mean abundance (*MA*) and mean intensity (*MI*) were calculated to reflect the infestations of the dominant rat hosts with *O. bacoti* mites. The patchiness index and Taylor’s power law were used to measure the spatial distribution of the mites. A total of 4121 *O. bacoti* mites were identified from 15 species of small mammal hosts in 27 of the 39 investigated counties, and 99.20% of them (4088/4121) were found on rodents. The majority of total *O. bacoti* mites was found in the flatland landscape (91.28%) and indoor habitat (73.48%). Moreover, 51.78% and 40.09% of *O. bacoti* mites were identified from *Rattus tanezumi* and *R. norvegicus*, the two synanthropic rat species. The mites had some host-specificity, with a preference to two dominant hosts (*R. tanezumi* and *R. norvegicus*), and they were of aggregated distribution on *R. tanezumi*.

**Abstract:**

(1) Background: As a species of gamasid mite, the tropical rat mite (*Ornithonyssus bacoti*) is a common ectoparasite on rodents and some other small mammals. Besides stinging humans to cause dermatitis, *O. bacoti* can be a vector of rickettsia pox and a potential vector of hemorrhagic fever with renal syndrome (HFRS). (2) Objective: The present study was conducted to understand the host selection of *O. bacoti* on different animal hosts and the distribution in different environmental gradients in Yunnan Province of Southwest China. (3) Methods: The original data came from the investigations in 39 counties of Yunnan, between 1990 and 2015. The animal hosts, rodents and some other small mammals were mainly trapped with mouse traps. The *O. bacoti* mites on the body surface of animal hosts were collected and identified in a conventional way. The constituent ratio (*Cr*), prevalence (*P_M_*), mean abundance (*MA*) and mean intensity (*MI*) were used to reflect infestations of animal hosts with *O. bacoti* mites. The patchiness index and Taylor’s power law were used to measure the spatial distribution pattern of *O. bacoti* mites on their hosts. (4) Results: A total of 4121 tropical rat mites (*O. bacoti*) were identified from 15 species and 14,739 individuals of hosts, and 99.20% of them were found on rodents. More than half of *O. bacoti* mites (51.78%) were identified from the Asian house rat (*Rattus tanezumi*), and 40.09% of the mites from the Norway rat (*R. norvegicus*) (*p* < 0.05). The infestations of *R. tanezumi* (*P_M_* = 7.61%, *MA* = 0.40 and *MI* = 5.31) and *R. norvegicus* (*P_M_* = 10.98, *MA* = 1.14 and *MI* = 10.39) with *O. bacoti* mites were significantly higher than those of other host species (*p* < 0.05). The infestations of two dominant rat hosts (*R. tanezumi* and *R. norvegicus*) with *O. bacoti* mites varied in different environmental gradients (latitudes, longitudes, altitudes, landscapes and habitats) and on different sexes and ages of the hosts. The prevalence of juvenile *R. norvegicus* rats with *O. bacoti* mites (*P_M_* = 12.90%) was significantly higher than that of adult rats (*P_M_* = 9.62%) (*p* < 0.05). The prevalence (*P_M_* = 38.46%) and mean abundance (*MA* = 2.28 mites/host) of *R. tanezumi* rats with *O. bacoti* mites in the high latitude were higher than those in the low latitudes (*p* < 0.05). The majority of the total collected 4121 *O. bacoti* mites was found in the flatland landscape (91.28%) and indoor habitat (73.48%) (*p* < 0.05). The *P_M_* (10.66%) and *MA* (0.49 mites/host) of *R. tanezumi* rats with *O. bacoti* mites were significantly higher in the indoor habitat than in the outdoor habitat (*p* < 0.05). The tropical rat mites showed an aggregated distribution pattern on their first dominant host, *R. tanezumi*. **Conclusion:** The tropical rat mite (*O. bacoti*) is a widely distributed species of gamasid mite in Yunnan Province, Southwest China, and its dominant hosts are two synanthropic species of rats, *R. tanezumi* and *R. norvegicus*. It is mainly distributed in the flatland landscape and indoor habitat. It has some host-specificity, with a preference to rodents, especially *R. tanezumi* and *R. norvegicus*. The *O. bacoti* mites are of aggregated distribution on *R. tanezumi* rats.

## 1. Introduction

As a worldwide species of gamasid mite, the tropical rat mite (*Ornithonyssus bacoti*) is widely distributed in nearly all parts of the world, except the Arctic and Antarctic regions [1,2]. It is a common ectoparasite on rodents (rats, mice and voles) and some other small mammals (e.g., insectivores and tree shrews), frequently occurring on the body surface and in the nests of its hosts. *Ornithonyssus bacoti* is the most important species of gamasid mites associated with medicine, and nearly all its stages (larvae, protonymphs, deutonymphs and adults) in the life cycle can invade and sting animal hosts for blood meal [3,4,5]. The dermatitis caused by the stinging of *O. bacoti* mites is frequently reported throughout the world [5,6,7,8,9,10,11,12,13,14,15,16]. Besides directly stinging humans, *O. bacoti* is associated with the transmission of some zoonoses. Together with another species of gamasid mite (*Allodermanyssus sanguineus*), the mite *O. bacoti* is the confirmed vector of rickettsia pox, caused by the pathogen *Rickettsia akari* [1,17,18]. Rickettsia pox is a zoonosis associated with rodents, and it is mainly prevalent in the United States, Canada, Russia, Ukraine, India and Egypt, etc. [17,19,20,21]. Besides being the intermediate host of the animal parasite *Litomosoides carinii*, the mite *O. bacoti* has been proved to be the potential vector of hemorrhagic fever with renal syndrome (HFRS) caused by hantavirus [22,23,24,25].

To date, there have been a lot of studies on the tropical rat mite, *O. bacoti*. Early on in the 1940s, some scholars began to feed *O. bacoti* in the laboratory and designed some special devices suitable for *O. bacoti* and some other sucking mites [26,27]. Many previous publications on *O. bacoti* and some other species of gamasid mites were about the mites’ ultrastructure [1,8,28,29,30], chromosome karyotype, gene sequencing, phylogeny and control [1,29,31,32,33,34], but only few studies were about the mite ecology. To date, there have been no systematically ecological studies on *O. bacoti* in Yunnan Province of Southwest China. Between 1990 and 2015, our research group made a long-term field investigation and accumulated abundant original data on gamasid mites in Yunnan. To take advantage of the previous investigation, the present paper analyzed the distribution and host selection of *O. bacoti* in Yunnan for the first time, which is an attempt to get more knowledge about *O. bacoti* and to enrich the ecological information of the mite.

## 2. Materials and Methods

### 2.1. Collection and Identification of Ornithonyssus bacoti and Its Animal Hosts

**Figure 1:**
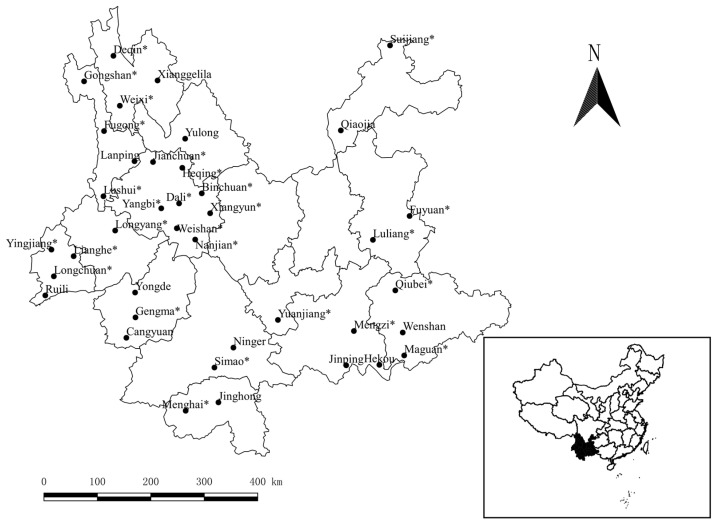
The 39 investigated counties and 27 counties with tropical rat mites (*Ornithonyssus bacoti*) collected (marked with “*”) in Yunnan Province, Southwest China (1990–2015).

The original data came from a long-term field investigation in 39 counties of Yunnan Province in Southwest China, between 1990 and 2015, and the investigated 39 counties were shown in Figure 1, in Results. A stratified sampling investigation was made in different geographical localities, latitudes, longitudes, altitude, landscapes and habitats. To capture animal hosts, mouse traps were placed in the indoor and outdoor habitats of each investigation site, in the evening, and then checked the following morning. The indoor habitats covered houses, barns, stables, etc., and the outdoor habitats covered paddy fields, cornfields, bush habitats, woodlands, etc. [35]. Each trapped host was euthanized through anesthesia with ether (cotton balls soaked with ether), within a closed container. Under the anesthesia, the gamasid mites on the body surface of each host were all collected and then preserved in Eppendorf tubes with 70% of ethanol. After the mite collection, each animal host was identified into species according to its morphological features [4,36,37]. Through the dehydration and clarification, the collected gamasid mites were mounted onto glass slides with Hoyer’s medium and they were then identified into species under microscopes [38,39]. Based on the identification of gamasid mites, the tropical rat mite (*O. bacoti*) was chosen as the target of the present study. In the animal euthanasia, most rodent pests in agriculture and forestry were anaesthetized to death because the local government encourages people to kill and eradicate them. Some non-pest small mammals (e.g., weasels, moles and some squirrels) were anaesthetized only for two to five minutes, according to their body size, and they were finally released to the wild when they woke up. The capturing of rodents and other small mammals was officially permitted by the local authority of wildlife service in Yunnan Province, China. The use of animals (including animal euthanasia) for research was officially approved by the Animals’ Ethics Committee of Dali University, and the permitted number was DLDXLL2020-1104. The specimens of voucher mites and representative animal hosts are deposited in the specimen repository of the Institute of Pathogens and Vectors, Dali University, Yunnan, China.

### 2.2. Infestation Statistics

In a conventional way, the constituent ratio (*Cr*), prevalence *(P_M_*), mean abundance (*MA*) and mean intensity (*MI*) were calculated to reflect the infestations of dominant hosts with tropical rat mites (*O. bacoti*) [4,40,41,42]. In the present study, *Cr* represents the percentage of *O. bacoti* mites, *P_M_* the percentage of infested hosts by the mites, *MA* the number of the mites on each examined host and *MI* the number of the mites on each infested host.

### 2.3. Analysis on Host Selection and Distribution

The infestations of dominant animal hosts with *O. bacoti* mites were compared on different sexes and ages of the hosts to reflect the host selection of the mites. The infestations were compared in different latitudes and longitudes, to reflect the mite’s horizontal distribution, and compared in different altitudes, to reflect the mite’s vertical distribution. The latitudes and longitudes were divided into three gradients, and the altitudes were divided into four gradients. The three latitude gradients include <24° N, 24° N–26° N and >26° N, and the four longitude gradients are <100° E, 100° E–102° E, 102° E–104° E and >104° E. The four altitude gradients are <1000 m, 1000–2000 m, 2001–3000 m and >3000 m.

### 2.4. Analysis of Spatial Distribution Pattern

The patchiness index (m*/m) and Taylor’s power were used to measure the spatial distribution pattern of tropical rat mites (*O. bacoti*) among different individuals of its dominant hosts [43,44]. The formulae of patchiness index and Taylor’s power are listed as follows.

(1) $$m*/m=\frac{m+(\frac{\sigma^{2}}{m}-1)}{m};\;\mathrm{lg}\sigma^{2}=\mathrm{lg}a+b\mathrm{lg}m$$

In the above formulae, m*/m = patchiness index, *m* = mean of *O. bacoti* mites on its dominant hosts and *σ^2^* = variance of the mites; lg *a* = intercept on the Y-axis, and *b* = the slope or regression coefficient. When m*/m > 1, *a* > 1 and *b* > 1 or *a* > 1, *b* = 1, the spatial distribution pattern is determined as the aggregated distribution; when m*/m = 1, *a* = 1 and *b* = 1, the random distribution; when m*/m < 1, *a* < 1, *b* < 1, the uniform (or even) distribution [45,46].

### 2.5. Analysis on Interspecific Association

Based on a contingency table (see Table 10 in Results), the association coefficient (*V*) was used to measure the interspecific association between the tropical rat mite (*O. bacoti*) and some other related species of gamasid mites on the dominant animal hosts. In the contingency table, *O. bacoti* was defined as “species X”, while the other related mite species was defined as “species Y”. The association coefficient (*V*) is as follows.

(2) $$V=\frac{ad-bc}{\sqrt{\left(a+b)(c+d)(a+c)(b+d\right)}}$$

In the above formula, *V* = the association coefficient between species X and Y; *a* = the host individuals on which species X and Y simultaneously occur; *b* = the host individuals on which species Y occurs, but species X does not occur; *c* = the host individuals on which species X occurs, but species Y does not occur; and *d* = the host individuals on which both species X and Y do not occur. When *V* > 0 and *p* < 0.05, the interspecific relationship between species X and Y is determined as the positive association; when *V* < 0 and *p* < 0.05, we have the negative association; P is the significance probability in Chi-square test (*χ^2^*).

### 2.6. Significance Test

Chi-square test (*χ^2^*) was used to test the significance of *Cr*, *P_M_* and *V*. Nonparametric test was used to test the significance of *MA* and *MI*. All the statistical analyses were performed with R software version 3.5.3.

## 3. Results

### 3.1. Abundance of Ornithonyssus bacoti

A total of 139,111 gamasid mites were collected from 74 species and 17,638 individuals of animal hosts, rodents and some other small mammals, in 39 counties of Yunnan. Of 139,111 collected gamasid mites, 137,210 of them were identified as 156 species and 39 genera in 13 families, and the remaining 1901 mites remained unidentified because of blemished, obscure and damaged structures or suspected new species. A total of 4121 tropical rat mites (*O. bacoti*) were identified from 15 species and 14,739 individuals of hosts, and they accounted for only 2.96% (4121/139,111) of the total mites. The identified 4121 *O. bacoti* mites were distributed in 27 counties (Figure 1).

### 3.2. Host Selection of Ornithonyssus bacoti

**Figure 2:**
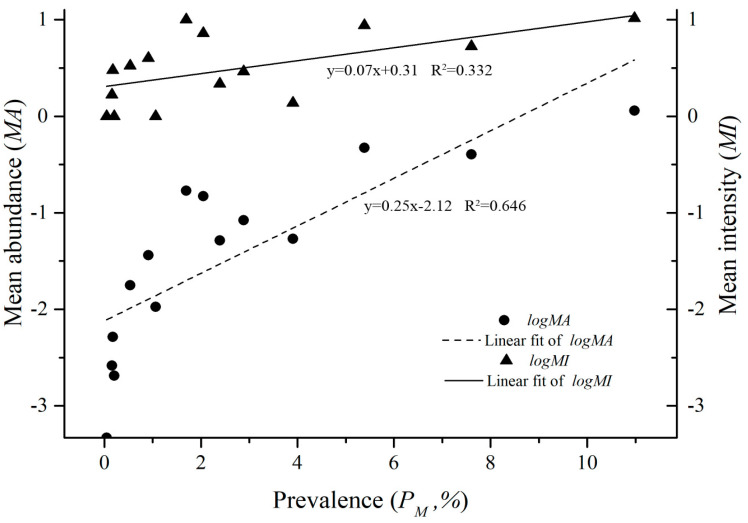
The linear regression between *P_M_*, *MA* and *MI* of 15 host species with *O. bacoti* mites in Yunnan Province, Southwest China (1990–2015).

The identified 4121 *O. bacoti* mites came from 15 species, 8 genera and 4 families of animal hosts in 3 orders, Rodentia, Soricomorpha and Scandetia. On the order level of animal hosts, 99.20% of *O. bacoti* mites (4088/4121) were found on the order Rodentia, which was significantly higher than that on Soricomorpha and Scandetia (*p* < 0.05). The percentages of *O. bacoti* mites found on the family Muridae (4088/4121 = 99.20%) and the genus *Rattus* (3953/4121 = 95.92%) were the highest of four host families and eight genera (*p* < 0.05). On the species level of hosts, the majority of *O. bacoti* mites was identified from two dominant rat hosts, with 51.78% of the mites on the Asian house rat (*Rattus tanezumi*) and 40.09% of the mites on the Norway rat (*R. norvegicus*) (*p* < 0.05). Of the 15 species of hosts, the infestations of the dominant rat hosts (*R. tanezumi* and *R. norvegicus*) with *O. bacoti* mites were significantly higher than those of other 13 species of hosts (*p* < 0.05) (Table 1). Positive linear correlations existed among *P_M_*, *MA* and *MI*, with *r* = 0.685 between *MA* and *MI*, *r* = 0.646 between *MA* and *P_M_* and *r* = 0.332 between *MI* and *PM* (*p* < 0.05) (Figure 2).

Different sexes and ages of two dominant rat hosts (*R. tanezumi* and *R. norvegicus*) showed some differences in the infestations with *O. bacoti* mites. The infestations of male rats (*R. tanezumi* and *R. norvegicus*) with *O. bacoti* mites were slightly higher than those of female rats, but the differences were of no statistical significance (*p* > 0.05) (Table 2 and Table 3). The prevalence of juvenile *R. norvegicus* rats with the mites (*P_M_* = 12.90%) was significantly higher than that of adult rats (*P_M_* = 9.62%) (*p* < 0.05). The mean abundance (*MA* mites/host) and mean intensity (*MI* mites/host) of juvenile *R. norvegicus* rats with the mites were slightly higher than those of adult rats, but the differences were of no statistical significance (*p* > 0.05). The infestations of juvenile *R. tanezumi* rats with the mites (*P_M_*, *MA* and *MI*) were also slightly higher than those of adult rats, but the differences were of no statistical significance (*p* > 0.05) (Table 2 and Table 3).

### 3.3. Horizontal Distribution of Ornithonyssus bacoti

The infestations of two dominant rat hosts (*R. tanezumi* and *R. norvegicus*) with *O. bacoti* mites showed some differences in different latitudes and longitudes (horizontal distribution). The *P_M_* (38.46%) and *MA* (2.28 mites/host) of *R. tanezumi* with *O. bacoti* mites, together with *MA* (2.04 mites/host) of *R. norvegicus* with the mites, were higher in the high latitude (>26° N) than in other latitudes (*p* < 0.05) (Table 4 and Table 5). The *MA* (0.63 mites/host) of *R. tanezumi* rats with the mites was higher in the longitude <100° E than in other three longitudes (*p* < 0.05), and the *P_M_* (16.81%) and *MA* (2.10 mites/host) of *R. norvegicus* with the mites were higher in the longitude 100° E–102° E than in other three longitudes (*p* < 0.05) (Table 4 and Table 5).

### 3.4. Vertical Distribution of Ornithonyssus bacoti

The infestations of two dominant rat hosts (*R. tanezumi* and *R. norvegicus*) with *O. bacoti* mites showed some differences in different altitudes (vertical distribution). The *P_M_* (27.27%) and *MA* (0.82 mites/host) of *R. tanezumi* rats with *O. bacoti* mites were highest above 3000 m, but *MI* (7.62 mites/host) was highest below 1000 m (*p* < 0.05). The *P_M_* (13.40%), *MA* (0.77 mites/host) and *MI* (5.74 mites/host) of *R. norvegicus* rats with the mites were highest at 1000–2000 m (*p* < 0.05) (Table 6).

### 3.5. Landscape and Habitat Distribution of Ornithonyssus bacoti

The majority of total collected 4121 *O. bacoti* mites was found in the flatland landscape (1894/2075 = 91.28%) and indoor habitat (3028/4121 = 73.48%) (*p* < 0.05). The infestations of two dominant rat hosts (*R. tanezumi* and *R. norvegicus*) with *O. bacoti* mites showed some differences in two kinds of landscapes (mountainous and flatland landscapes), but the differences were of no statistical significance (*p* > 0.05) (Table 7). The infestations of *R. tanezumi* and *R. norvegicus* with the mites also showed some differences in two kinds of habitats. The *P_M_* (10.66%) and *MA* (0.49 mites/host) of *R. tanezumi* rats with the mites were significantly higher in the indoor habitat than in the outdoor habitat (*p* < 0.05). The *P_M_* (11.50%), *MA* (1.30 mites/host) and *MI* (11.31 mites/host) of *R. norvegicus* rats with the mites were higher in the indoor habitat than in the outdoor habitat, but the differences were of no statistical significance (*p* > 0.05) (Table 8).

### 3.6. Spatial Distribution Pattern and Interspecific Association

A total of 5285 Asian house rats (*R. tanezumi*), the first dominant host of the tropical rat mites (*O. bacoti*), were captured in 35 of 39 investigated counties, but there were only 24 counties where *R. tanezumi* harbored *O. bacoti* mites. To establish a linear regression equation based on Taylor’s power law, the 24 counties were recombined as four “sample units”, according to their adjacent locations, and then the mean (*m*) and variance (*σ^2^*) of *O. bacoti* mites on *R. tanezumi* rats in each sample unit were calculated (Table 9). According to the calculated *m* and *σ^2^*, a linear regression equation was established as lg *σ^2^* = lg 39.30 + 1.42 lg *m*, where both *a* (39.30) and *b* (1.42) were beyond 1 (*a* > 1, *b* > 1), the border value for determining the aggregated distribution. The calculated patchiness index (m*/m) in each sample unit was also higher than 1 (m*/m > 1), the border value for determining the aggregated distribution (Table 9). On the body surface of *R. tanezumi* rats, there were a lot of *L. nuttalli* mites (the other species of gamasid mites) that co-occurred with *O. bacoti* mites, and therefore the interspecific association between *O. bacoti* and *L. nuttalli* was studied. The result showed that there was a slight negative association between *O. bacoti* and *L. nuttalli* (*V* = −0.0794, *V* < 0, *p* < 0.05) (Table 10).

## 4. Discussion

In laboratories, the tropical rat mite (*O. bacoti*) is often found on the experimental rats and mice, and it does a great harm to experimental animals [6,47,48]. Therefore, it is important to make a systematic study on *O. bacoti*. Some previous ecological studies of gamasid mites mainly focused on some local species surveys, faunal studies and community investigations [4,36,49]. Although some local investigations on the fauna and community of gamasid mites included the constituent ratio of *O. bacoti*, there were few independent and systematic studies of *O. bacoti* [16,38,50]. The present study systematically analyzed the distribution and host selection of *O. bacoti* in Yunnan Province of Southwest China for the first time. The original data came from a long-term investigation between 1990 and 2015, and the investigated 39 counties covered the different localities of Yunnan Province, Southwest China. The tropical rat mite (*O. bacoti*) was found in 27 of 39 investigated counties (Figure 1), and it suggests that *O. bacoti* is a widely distributed species of gamasid mites in Yunnan.

The present study showed that 99.20% of tropical rat mites (*O. bacoti*) were found on rodents (the order Rodentia), even though three orders of hosts (Rodentia, Soricomorpha and Scandetia) harbored the mites. Although *O. bacoti* mites occurred on different categories of hosts (15 species, 8 genera, 4 families and 3 orders), most of them were identified from two dominant rat species, the Asian house rat (*R. tanezumi*) and the Norway rat or brown rat (*R. norvegicus*). The infestations of *R. tanezumi* and *R. norvegicus* with *O. bacoti* mites were significantly higher than those of other 13 host species. The results suggest that *O. bacoti* has some host-specificity and it has a preference to *R. tanezumi* and *R. norvegicus* in Yunnan. The higher prevalence (*P_M_*) of juvenile *R. norvegicus* rats with *O. bacoti* mites than that of adult rats (Table 2) may imply the preference of the mites to juvenile hosts. Rodents are closely related to human beings, and they are the infection source and reservoir hosts of many zoonotic diseases [18,51]. The rodent-preference of *O. bacoti* would increase the potential risk of the mite’s attacking humans and spreading some zoonoses. The Asian house rat (*R. tanezumi*) and the Norway rat (*R. norvegicus*) are two major species of rodents associated with human settlements in Yunnan Province and some other places of China [52,53]. *Rattus tanezumi* (often called *R. flavipectus* in China) is widely distributed in the vast areas south of the Yangtze River, in Southern China. It is a very common rodent species in residential areas (especially the indoor habitats) in Central and Southern Yunnan [54,55]. *Rattus norvegicus* is widely distributed in the whole China, and it is also a very common rodent species in residential areas (especially the indoor habitats) in Yunnan, often co-occurring in the same areas with *R. tanezumi* [35,56,57,58]. The previous studies revealed that the main hosts of *O. bacoti* included some synanthropic rats and mice with humans (especially *R. norvegicus*) and experimental rats and mice [3,7,18]. The most commonly used laboratory rat is descended from *R. norvegicus*, which retains many biological characteristics of its ancestor *R. norvegicus* [59,60]. The frequent occurrence of *O. bacoti* on *R. tanezumi* and *R. norvegicus* would highly increase the risk of the mites’ attacking humans and spreading some zoonoses.

*Rattus tanezumi* and *R. norvegicus* were two dominant rat hosts of *O. bacoti* mites, and therefore the present paper analyzed the infestations of the two host species with the mites in different horizontal gradients (latitudes and longitudes) and vertical gradients (altitudes). The results showed that the infestations of the rats (*R. tanezumi* and *R. norvegicus*) with *O. bacoti* mites showed some differences in different horizontal and vertical gradients. Some infestation indices (*P_M_* and *MA*) were higher in the high latitude (>26° N) and low longitudes (<100° E and 100° E–102° E) than in other latitudes and longitudes (Table 4 and Table 5). The *P_M_* and *MA* of *R. tanezumi* rats with the mites were highest above 3000 m, but *MI* was highest below 1000 m. The *P_M_*, *MA* and *MI* of *R. norvegicus* rats with the mites were highest at 1000–2000 m (Table 6). The results indicated an unstable fluctuation in different vertical gradients. The climates in Yunnan province greatly vary from region to region because of complex topography and altitude gradients. Even within the same latitude or longitude gradient zone, the climate at a mountainous site with higher altitude may be very different from that at a flatland site with lower altitude [61,62,63]. The different infestations of the rats (*R. tanezumi* and *R. norvegicus*) with *O. bacoti* mites in different horizontal and vertical gradients may be related to different climates (temperature, humidity and rainfall, etc.) in different geographical localities. However, it is difficult to explain the unstable fluctuation of the mite infestations in different horizontal and vertical gradients, and more research studies still remain to be conducted.

Located in the southwest of China, Yunnan is a mountainous province where mountainous landscapes with higher altitude and lower temperature account for 84% of the whole territory, and flatland landscapes with lower altitude and higher temperature are often embedded in mountainous landscapes [63,64]. Although the flatland landscape only takes a small portion of the whole territory, the majority of *O. bacoti* mites (91.28%) was found in the flatland landscape, and this suggests that *O. bacoti* is mainly distributed in the flatland landscape. The infestations of the rats (*R. tanezumi* and *R. norvegicus*) with the mites showed some differences in mountainous and flatland landscapes, but the differences were of no statistical significance (Table 7). In habitat distribution, 73.48% of *O. bacoti* mites were collected in the indoor habitat. The *P_M_* and *MA* of *R. tanezumi* rats with the mites were significantly higher in the indoor habitat than in the outdoor habitat (Table 8), indicating the preference of the mites for the indoor habitat. The previous study showed that the optimum temperature for the development of *O. bacoti* was about 25 °C ± 5 °C, and higher than 30 °C or lower than 20 °C was not suitable for the mites’ development [65]. The outdoor habitat in the present study involved a series of different sub-habitats, or microhabitats, such as cultivated farmlands (e.g., paddy fields and cornfields) and uncultivated bush areas and woodlands; the micro-climates in the outdoor habitat are often unstable. In comparison with the outdoor habitat, the indoor habitat is a relatively closed environment with a relatively stable and warm temperature and low humidity [65,66]. The stable and warm micro-climate with relatively low humidity in the indoor habitat may be more suitable to the growth and reproduction of *O. bacoti* mites. The frequent occurrence of *O. bacoti* in the indoor habitat would highly increase the risk of the mites’ invading and stinging humans. When rats and mice are not available for *O. bacoti* mites to suck the blood of, the mites in the indoor habitat may quickly move onto humans for the blood meal and then expand their range of activity [1,67,68].

The measurement of spatial distribution pattern of a certain population is one of important issues in arthropod ecology [69,70]. There are usually three types of spatial distribution patterns: uniform (or even) distribution, random distribution and aggregated distribution [52,71,72,73]. There are a variety of statistical methods to measure the spatial distribution pattern of a certain population, and the patchiness index and Taylor’s power law are two of them [43,44,74]. According to the statistics of the patchiness index and Taylor’s power law, tropical rat mites (*O. bacoti*) were determined to be of aggregated distribution on *R. tanezumi*, the first dominant host. The aggregated distribution indicates that the mite infestation is not even among different hosts. Some hosts may harbor a large number of mites, forming a clump of mites on their body surface, while some other hosts may have no or very few mites on their body surface. The aggregated distribution pattern of *O. bacoti* in the present study is consistent with that of some other ectoparasites, such as chigger mites; this is a common phenomenon in many parasites [38,52,71]. The aggregated distribution may be beneficial to the survival, mating and defense of the parasites [4,38,72,73].

The analysis of the interspecific relationship between any two different species is also an important issue in animal ecology [75,76]. The association coefficient (*V*) used in the present study is a simple way to measure the interspecific relationship between any two species [77,78,79,80]. The negative value of the association coefficient (*V* = −0.0794) may imply that there is a slight negative association between *O. bacoti* and *L. nuttalli*, but the value of “*V* = −0.0794” was very close to “0”, and more research is still needed.

## 5. Conclusions

The tropical rat mite (*O. bacoti*) is a widely distributed species of gamasid mite in Yunnan Province, Southwest China, and its dominant hosts are two synanthropic species of rats, *R. tanezumi* and *R. norvegicus*. It is mainly distributed in the flatland landscape and indoor habitat. It has some host-specificity, with a preference to rodents, especially *R. tanezumi* and *R. norvegicus*. The *O. bacoti* mites are of aggregated distribution on *R. tanezumi* rats.

## Figures and Tables

**Table 1:** Infestations of two dominant rat hosts (*Rattus tanezumi* and *R. norvegicus*) with tropical rat mites (*Ornithonyssus bacoti*) in Yunnan Province, Southwest China (1990–2015).

Host Species	Host Individuals		Infestations of Hosts with *O. bacoti* Mites				
	Examined Hosts	Infested Hosts	Mite Individuals	Constituent Ratios of Mites (*Cr*, %)	Prevalence (*P_M_*, %)	Mean Abundance (*MA*)	Mean Intensity (*MI*)
*Rattus tanezumi*	5285	402	2134	51.78	7.61	0.40	5.31
*Rattus norvegicus*	1448	159	1652	40.09	10.98	1.14	10.39
Other 13 host species	8006	68	335	8.13	0.85	0.04	4.93
Total *	14,739	629	4121	100.00	4.27	0.28	6.55

*Annotation: The other 13 species of animal hosts are *Rattus nitidus*, *Mus musculus*, *R. brunneusculus (R. sladeni)*, *Suncus murinus*, *Crocidura attenuata*, *Apodemus draco*, *Niviventer andersoni*, *Tupaia belangeri*, *A. chevrieri*, *N. confucianus*, *Eothenomys miletus*, *M. caroli and A. peninsulae*.

**Table 2:** Infestations of different sexes and ages of *R. tanezumi* rats with *O. bacoti* mites in Yunnan Province, Southwest China (1990–2015).

Sexes and Ages of Hosts	Examined Hosts	Infested Hosts	Infestations of Different Sexes and Ages of Rat Hosts with *O. bacoti* Mites				
			Mite Individuals	Constituent Ratios of the Mites (*Cr*, %)	Prevalence (*P_M_*, %)	Mean Abundance (*MA*)	Mean Intensity (*MI*)
Males	2540	207	1157	72.54	8.15	0.46	5.59
Females	2534	194	974	61.07	7.66	0.38	5.02
Total *	5074	401	2131	100.00	7.90	0.42	5.31
Juveniles	1227	99	536	25.15	8.07	0.44	5.41
Adults	3847	302	1595	74.85	7.85	0.41	5.28
Total *	5074	401	2131	100.00	7.90	0.42	5.31

* Annotation: The animal hosts without records of sexes and ages were not included in the above table.

**Table 3:** Infestations of different sexes and ages of *R. norvegicus* rats with *O. bacoti* mites in Yunnan Province, Southwest China (1990–2015).

Sexes and Ages of Hosts	Examined Hosts	Infested Hosts	Infestations of Different Sexes and Ages of Rat Hosts with *O. bacoti* Mites				
			Mite Individuals	Constituent Ratios of the Mites (*Cr*, %)	Prevalence (*P_M_*, %)	Mean Abundance (*MA*)	Mean Intensity (*MI*)
Males	734	82	919	55.63	11.17	1.25	11.21
Females	712	77	733	44.37	10.81	1.03	9.52
Total *	1446	159	1652	100.00	11.00	1.14	10.39
Juveniles	659	85	1191	72.09	12.90	1.81	14.01
Adults	769	74	461	27.91	9.62	0.60	6.23
Total *	1428	159	1652	100.00	11.13	1.16	10.39

* Annotation: The animal hosts without records of sexes and ages were not included in the above table.

**Table 4:** Infestations of *R. tanezumi* rats with *O. bacoti* mites in different latitudes and longitudes of Yunnan Province, Southwest China (1990–2015).

Latitudes and Longitudes	Examined Hosts	Infested Hosts	Infestations of Rat Hosts with *O. bacoti* Mites in Different Latitudes and Longitudes				
			Mite Individuals	Constituent Ratios of the Mites (*Cr*, %)	Prevalence (*P_M_*, %)	Mean Abundance (*MA*)	Mean Intensity (*MI*)
Low latitude <24° N	2967	73	345	16.17	2.46	0.12	4.73
Middle latitude 24–26° N	2279	314	1700	79.66	13.78	0.75	5.41
High latitude >26° N	39	15	89	4.17	38.46	2.28	5.93
Total *	5285	402	2134	100	7.61	0.40	5.31
Longitude <100° E	1789	185	1136	53.23	10.34	0.63	6.14
Longitude 100° E–102° E	3164	193	920	43.11	6.10	0.29	4.77
Longitude 102° E–104° E	205	8	18	0.84	3.90	0.09	2.25
Longitude >104° E	127	16	60	2.81	12.60	0.47	3.75
Total *	5285	402	2134	100	7.61	0.40	5.31

* Annotation: The animal hosts without records of latitudes and longitudes were not included in the above table.

**Table 5:** Infestations of *R. norvegicus* rats with *O. bacoti* mites in different latitudes and longitudes of Yunnan Province, Southwest China (1990–2015).

Latitudes and Longitudes	Examined Hosts	Infested Hosts	Infestations of Rat Hosts with *O. bacoti* Mites in Different Latitudes and Longitudes				
			Mite Individuals	Constituent Ratios of the Mites (*Cr*, %)	Prevalence (*P_M_*, %)	Mean Abundance (*MA*)	Mean Intensity (*MI*)
Low latitude <24° N	117	8	25	1.51	6.84	0.21	3.13
Middle latitude 24–26° N	1264	141	1490	90.19	11.16	1.18	10.57
High latitude >26° N	67	10	137	8.29	14.93	2.04	13.70
Total *	1448	159	1652	100.00	10.98	1.14	10.39
Longitude <100° E	200	17	149	9.02	8.50	0.75	8.76
Longitude 100° E–102° E	690	116	1447	87.59	16.81	2.10	12.47
Longitude 102° E–104° E	259	25	53	3.21	9.65	0.20	2.12
Longitude >104° E	299	1	3	0.18	0.33	0.01	3.00
Total *	1448	159	1652	100	10.98	1.14	10.39

* Annotation: The animal hosts without records of latitudes and longitudes were not included in the above table.

**Table 6:** Infestations of *R. tanezumi* and *R. norvegicus* rats with *O. bacoti* mites in different altitudes of Yunnan Province, Southwest China (1990–2015).

Dominant Rat Hosts and Altitudes (Meters)	Examined Hosts	Infested Hosts	Infestations of Rat Hosts with *O. bacoti* mites in Different Altitudes				
			Mite Individuals	Constituent Ratios of the Mites (*Cr*, %)	Prevalence (*P_M_*, %)	Mean Abundance (*MA*)	Mean Intensity (*MI*)
***R. tanezumi***							
<1000	932	94	716	47.32	10.09	0.77	7.62
1000–2000	1548	210	763	50.43	13.57	0.49	3.63
2001–3000	195	13	25	1.65	6.67	0.13	1.92
>3000	11	3	9	0.59	27.27	0.82	3.00
Total *	2686	320	1513	100.00	11.91	0.56	4.73
***R. norvegicus***							
<1000	15	0	0	0	0	0	-
1000–2000	291	39	224	85.82	13.40	0.77	5.74
2001–3000	358	23	37	14.18	6.42	0.10	1.61
>3000	28	0	0	0	0	0	-
Total *	692	62	261	100.00	8.96	0.38	4.21

* Annotation: The animal hosts without records of altitudes were not included in the above table.

**Table 7:** Infestations of *R. tanezumi* and *R. norvegicus* rats with *O. bacoti* mites in different landscapes of Yunnan Province, Southwest China (1990–2015).

Dominant Rat Hosts and Landscapes	Examined Hosts	Infested Hosts	Infestations of Rat Hosts with *O. bacoti* Mites in Different Landscapes				
			Mite Individuals	Constituent Ratios of the Mites (*Cr*, %)	Prevalence (*P_M_*, %)	Mean Abundance (*MA*)	Mean Intensity (*MI*)
***R. tanezumi***							
Flatland landscape	2094	63	487	81.03	3.01	0.23	7.73
Mountainous landscape	879	31	114	18.97	3.53	0.13	3.68
Total *	2973	94	601	100.00	3.16	0.20	6.39
***R. norvegicus***							
Flatland landscape	940	104	1282	100.00	11.06	1.36	12.33
Mountainous landscape	110	0	0	0	0	0	-
Total *	1050	104	1282	100.00	9.90	1.22	12.33

* Annotation: The animal hosts without records of landscapes were not included in the above table.

**Table 8:** Infestations of *R. tanezumi* and *R. norvegicus* rats with *O. bacoti* mites in different habitats of Yunnan Province, Southwest China (1990–2015).

Dominant Rat Hosts and Habitats	Examined Hosts	Infested Hosts	Infestations of Rat Hosts with *O. bacoti* Mites in Different Habitats				
			Mite Individuals	Constituent Ratios of the Mites (*Cr*, %)	Prevalence (*P_M_*, %)	Mean Abundance (*MA*)	Mean Intensity (*MI*)
***R. tanezumi***							
Indoor habitats	2607	278	1274	59.70	10.66	0.49	4.58
Outdoor habitats	2678	124	860	40.30	4.63	0.32	6.94
Total *	5285	402	2134	100.00	7.61	0.40	5.31
***R. norvegicus***							
Indoor habitats	1191	137	1549	93.77	11.50	1.30	11.31
Outdoor habitats	241	22	103	6.23	9.13	0.43	4.68
Total *	1432	159	1652	100.00	11.10	1.15	10.39

* Annotation: The animal hosts without records of habitats were not included in the above table.

**Table 9:** The mean (*m*), variance (*σ^2^*) and patchiness index (m*/m) of *O. bacoti* mites on *R. tanezumi* rats in each recombined sample unit of Yunnan, Southwest China (1990–2015).

Sample Units	Individuals of *R. tanezumi* Rats	Individuals of *O. bacoti* Mites	Mean (*m*)	Variance (*σ^2^*)	Patchiness index (m*/m)
1	1071	871	0.81	68.98	104.06
2	235	740	3.15	106.28	11.40
3	1329	375	0.28	7.66	93.65
4	2650	148	0.06	0.45	126.36
Total	5285	2134	-	-	-

Annotation: Each sample unit represents the following counties: 1 = Njian + Dali + Binchuan +Yangbi + Xiangyun + Weishan + Jianchuan + Heqing; 2 = Lushui + Fugong + Weixi + Gongshan + Lijiang; 3 = Gengma + Lianghe + Longchuan + Yingjiang + Longyang + Ruili + Yongde + Cangyuan; 4 = Luliang + Fuyuan + Maguan + Suijiang + Menghai + Yuanjiang + Simao + Mengzi + Jinghong + Ninger + Jinping + Hekou + Qiubei + Wenshan.

**Table 10:** The contingency table for measuring the interspecific association between *O. bacoti* mites and *L. nuttalli* mites on the body surface of *R. tanezumi rats* in Yunnan, Southwest China (1990–2015).

Items		*O. bacoti* Mites (Species X)		Total
		+	−	
*L. nuttalli* mites	+	79 (*a*)	1646 (*b*)	1725 (*a* + *b*)
(Species Y)	−	323 (*c*)	3237 (*d*)	3560 (*c* + *d*)
Total		402 (*a* + *c*)	4883 (*b* + *d*)	5285 (*n*)

## Data Availability

The experimental data used to support the findings of this study are available from the corresponding author request.
